# Supplementary material for: Iterative improvement in the automatic modular design of robot swarms
Source: PeerJ Comput Sci. 2020 Dec 7;6:e322. doi: 10.7717/peerj-cs.322 (PMC7924708; doi:10.7717/peerj-cs.322)
Supplement: Supplemental Information 3 [file peerj-cs-06-322-s003.zip › argos3/doc/api/standalone/a00650_source.html]

ARGoS: plugins/simulator/visualizations/qt-opengl/qtopengl\_lua\_statetree\_model.cpp Source File


- Main Page
- Related Pages
- Namespaces
- Classes
- Files

- File List
- File Members

# plugins/simulator/visualizations/qt-opengl/qtopengl\_lua\_statetree\_model.cpp

Go to the documentation of this file.

```
00001 
00007 #include "qtopengl_lua_statetree_model.h"
00008 #include "qtopengl_lua_statetree_item.h"
00009 
00010 #include <argos3/core/utility/logging/argos_log.h>
00011 #include <argos3/core/wrappers/lua/lua_utility.h>
00012 
00013 namespace argos {
00014 
00015    /****************************************/
00016    /****************************************/
00017 
00018    CQTOpenGLLuaStateTreeModel::CQTOpenGLLuaStateTreeModel(lua_State* pt_state,
00019                                                           bool b_remove_empty_tables,
00020                                                           QObject* pc_parent) :
00021       QAbstractItemModel(pc_parent),
00022       m_ptState(pt_state),
00023       m_bRemoveEmptyTables(b_remove_empty_tables) {
00024       m_pcDataRoot = new CQTOpenGLLuaStateTreeItem();
00025    }
00026 
00027    /****************************************/
00028    /****************************************/
00029 
00030    CQTOpenGLLuaStateTreeModel::~CQTOpenGLLuaStateTreeModel() {
00031       delete m_pcDataRoot;
00032    }
00033 
00034    /****************************************/
00035    /****************************************/
00036 
00037    QVariant CQTOpenGLLuaStateTreeModel::data(const QModelIndex& c_index,
00038                                              int n_role) const {
00039       if(!c_index.isValid()) {
00040          return QVariant();
00041       }
00042       if(n_role != Qt::DisplayRole) {
00043          return QVariant();
00044       }
00045       CQTOpenGLLuaStateTreeItem* pcItem = static_cast<CQTOpenGLLuaStateTreeItem*>(c_index.internalPointer());
00046       return pcItem->GetData(c_index.column());
00047    }
00048 
00049    /****************************************/
00050    /****************************************/
00051 
00052    Qt::ItemFlags CQTOpenGLLuaStateTreeModel::flags(const QModelIndex& c_index) const {
00053       if (!c_index.isValid()) {
00054          return 0;
00055       }
00056       else {
00057          return Qt::ItemIsEnabled;
00058       }
00059    }
00060 
00061    /****************************************/
00062    /****************************************/
00063 
00064    QModelIndex CQTOpenGLLuaStateTreeModel::index(int n_row,
00065                                                  int n_column,
00066                                                  const QModelIndex& c_parent) const {
00067       if(!hasIndex(n_row, n_column, c_parent)) {
00068          return QModelIndex();
00069       }
00070       CQTOpenGLLuaStateTreeItem* pcParentItem;
00071       if(!c_parent.isValid()) {
00072          pcParentItem = m_pcDataRoot;
00073       }
00074       else {
00075          pcParentItem = static_cast<CQTOpenGLLuaStateTreeItem*>(c_parent.internalPointer());
00076       }
00077       CQTOpenGLLuaStateTreeItem* pcChildItem = pcParentItem->GetChild(n_row);
00078       if(pcChildItem) {
00079          return createIndex(n_row, n_column, pcChildItem);
00080       }
00081       else {
00082          return QModelIndex();
00083       }
00084    }
00085 
00086    /****************************************/
00087    /****************************************/
00088 
00089    QModelIndex CQTOpenGLLuaStateTreeModel::parent(const QModelIndex& c_index) const {
00090       if (!c_index.isValid()) {
00091          return QModelIndex();
00092       }
00093       CQTOpenGLLuaStateTreeItem* pcChildItem = static_cast<CQTOpenGLLuaStateTreeItem*>(c_index.internalPointer());
00094       CQTOpenGLLuaStateTreeItem* pcParentItem = pcChildItem->GetParent();
00095       if (pcParentItem == m_pcDataRoot) {
00096          return QModelIndex();
00097       }
00098       else {
00099          return createIndex(pcParentItem->GetRow(), 0, pcParentItem);
00100       }
00101    }
00102 
00103    /****************************************/
00104    /****************************************/
00105 
00106    int CQTOpenGLLuaStateTreeModel::rowCount(const QModelIndex& c_parent) const {
00107       CQTOpenGLLuaStateTreeItem* pcParentItem;
00108       if(c_parent.column() > 0) {
00109          return 0;
00110       }
00111       if(!c_parent.isValid()) {
00112          pcParentItem = m_pcDataRoot;
00113       }
00114       else {
00115          pcParentItem = static_cast<CQTOpenGLLuaStateTreeItem*>(c_parent.internalPointer());
00116       }
00117       return pcParentItem->GetNumChildren();
00118    }
00119 
00120    /****************************************/
00121    /****************************************/
00122 
00123    void CQTOpenGLLuaStateTreeModel::SetLuaState(lua_State* pt_state) {
00124       m_ptState = pt_state;
00125       Refresh();
00126    }
00127 
00128    /****************************************/
00129    /****************************************/
00130 
00131    void CQTOpenGLLuaStateTreeModel::Refresh() {
00132       beginResetModel();
00133       delete m_pcDataRoot;
00134       m_pcDataRoot = new CQTOpenGLLuaStateTreeItem();
00135       lua_pushnil(m_ptState);
00136       lua_getglobal(m_ptState, "_G");
00137       ProcessLuaState(m_ptState, m_pcDataRoot);
00138       m_pcDataRoot->SortChildren();
00139       lua_pop(m_ptState, 2);
00140       endResetModel();
00141    }
00142 
00143    /****************************************/
00144    /****************************************/
00145 
00146    void CQTOpenGLLuaStateTreeModel::Refresh(int) {
00147       Refresh();
00148    }
00149 
00150    /****************************************/
00151    /****************************************/
00152 
00153    void CQTOpenGLLuaStateTreeModel::ProcessLuaState(lua_State* pt_state,
00154                                                     CQTOpenGLLuaStateTreeItem* pc_item) {
00155       QList<QVariant> cData;
00156       switch(lua_type(pt_state, -2)) {
00157          case LUA_TBOOLEAN:
00158             cData << lua_toboolean(pt_state, -2);
00159             break;
00160          case LUA_TNUMBER:
00161             cData << lua_tonumber(pt_state, -2);
00162             break;
00163          case LUA_TSTRING:
00164             cData << lua_tostring(pt_state, -2);
00165             break;
00166          default: break;
00167       }
00168       if(lua_istable(pt_state, -1)) {
00169          CQTOpenGLLuaStateTreeItem* pcChild = new CQTOpenGLLuaStateTreeItem(cData, pc_item);
00170          pc_item->AddChild(pcChild);
00171          lua_pushnil(pt_state);
00172          while(lua_next(pt_state, -2)) {
00173             if(IsTypeVisitable(pt_state)) {
00174                ProcessLuaState(pt_state, pcChild);
00175             }
00176             lua_pop(pt_state, 1);
00177          }
00178          if(m_bRemoveEmptyTables) {
00179             if(pcChild->GetNumChildren() == 0) {
00180                pc_item->RemoveChild(pcChild);
00181             }
00182          }
00183       }
00184       else {
00185          switch(lua_type(pt_state, -1)) {
00186             case LUA_TBOOLEAN:
00187                cData << lua_toboolean(pt_state, -1);
00188                pc_item->AddChild(new CQTOpenGLLuaStateTreeItem(cData, pc_item));
00189                break;
00190             case LUA_TNUMBER:
00191                cData << lua_tonumber(pt_state, -1);
00192                pc_item->AddChild(new CQTOpenGLLuaStateTreeItem(cData, pc_item));
00193                break;
00194             case LUA_TSTRING:
00195                cData << lua_tostring(pt_state, -1);
00196                pc_item->AddChild(new CQTOpenGLLuaStateTreeItem(cData, pc_item));
00197                break;
00198             case LUA_TFUNCTION:
00199                cData[0] = cData[0].toString() + tr("()");
00200                pc_item->AddChild(new CQTOpenGLLuaStateTreeItem(cData, pc_item));
00201                break;
00202             default:
00203                break;
00204          }
00205       }
00206    }
00207 
00208    /****************************************/
00209    /****************************************/
00210    
00211    CQTOpenGLLuaStateTreeVariableModel::CQTOpenGLLuaStateTreeVariableModel(lua_State* pt_state,
00212                                                                           bool b_remove_empty_tables,
00213                                                                           QObject* pc_parent) :
00214       CQTOpenGLLuaStateTreeModel(pt_state, b_remove_empty_tables, pc_parent) {}
00215 
00216    /****************************************/
00217    /****************************************/
00218 
00219    QVariant CQTOpenGLLuaStateTreeVariableModel::headerData(int n_section,
00220                                                            Qt::Orientation e_orientation,
00221                                                            int n_role) const {
00222       if(e_orientation != Qt::Horizontal ||
00223          n_role != Qt::DisplayRole ||
00224          n_section > 1) {
00225          return QVariant();
00226       }
00227       else {
00228          return n_section == 0 ? tr("Variable") : tr("Value");
00229       }
00230    }
00231 
00232    /****************************************/
00233    /****************************************/
00234 
00235    int CQTOpenGLLuaStateTreeVariableModel::columnCount(const QModelIndex&) const {
00236       return 2;
00237    }
00238 
00239    /****************************************/
00240    /****************************************/
00241 
00242    bool CQTOpenGLLuaStateTreeVariableModel::IsTypeVisitable(lua_State* pt_state) {
00243       int nValueType = lua_type(pt_state, -1);
00244       int nKeyType = lua_type(pt_state, -2);
00245       if(nValueType == LUA_TSTRING || nValueType == LUA_TNUMBER || nValueType == LUA_TBOOLEAN) {
00246          if(nKeyType != LUA_TSTRING) {
00247             return true;
00248          }
00249          else if(nKeyType == LUA_TSTRING) {
00250             return std::string(lua_tostring(pt_state, -2)) != "_VERSION";
00251          }
00252       }
00253       else if(nValueType == LUA_TTABLE) {
00254          if(nKeyType == LUA_TNUMBER) {
00255             return true;
00256          }
00257          else if(nKeyType == LUA_TSTRING) {
00258             return 
00259                std::string(lua_tostring(pt_state, -2)) != "_G"        &&
00260                std::string(lua_tostring(pt_state, -2)) != "coroutine" &&
00261                std::string(lua_tostring(pt_state, -2)) != "debug"     &&
00262                std::string(lua_tostring(pt_state, -2)) != "io"        &&
00263                std::string(lua_tostring(pt_state, -2)) != "os"        &&
00264                std::string(lua_tostring(pt_state, -2)) != "package"   &&
00265                std::string(lua_tostring(pt_state, -2)) != "string"    &&
00266                std::string(lua_tostring(pt_state, -2)) != "table";
00267          }
00268       }
00269       return false;
00270    }
00271 
00272    /****************************************/
00273    /****************************************/
00274    
00275    CQTOpenGLLuaStateTreeFunctionModel::CQTOpenGLLuaStateTreeFunctionModel(lua_State* pt_state,
00276                                                                           bool b_remove_empty_tables,
00277                                                                           QObject* pc_parent) :
00278       CQTOpenGLLuaStateTreeModel(pt_state, b_remove_empty_tables, pc_parent) {}
00279 
00280    /****************************************/
00281    /****************************************/
00282 
00283    QVariant CQTOpenGLLuaStateTreeFunctionModel::headerData(int n_section,
00284                                                            Qt::Orientation e_orientation,
00285                                                            int n_role) const {
00286       return QVariant();
00287    }
00288 
00289    /****************************************/
00290    /****************************************/
00291 
00292    int CQTOpenGLLuaStateTreeFunctionModel::columnCount(const QModelIndex&) const {
00293       return 1;
00294    }
00295 
00296    /****************************************/
00297    /****************************************/
00298 
00299    bool CQTOpenGLLuaStateTreeFunctionModel::IsTypeVisitable(lua_State* pt_state) {
00300       int nValueType = lua_type(pt_state, -1);
00301       int nKeyType = lua_type(pt_state, -2);
00302       if(nValueType == LUA_TFUNCTION && nKeyType == LUA_TSTRING) {
00303             return 
00304                std::string(lua_tostring(pt_state, -2)) != "assert" &&
00305                std::string(lua_tostring(pt_state, -2)) != "collectgarbage" &&
00306                std::string(lua_tostring(pt_state, -2)) != "dofile" &&
00307                std::string(lua_tostring(pt_state, -2)) != "error" &&
00308                std::string(lua_tostring(pt_state, -2)) != "gcinfo" &&
00309                std::string(lua_tostring(pt_state, -2)) != "getfenv" &&
00310                std::string(lua_tostring(pt_state, -2)) != "getmetatable" &&
00311                std::string(lua_tostring(pt_state, -2)) != "ipairs" &&
00312                std::string(lua_tostring(pt_state, -2)) != "load" &&
00313                std::string(lua_tostring(pt_state, -2)) != "loadfile" &&
00314                std::string(lua_tostring(pt_state, -2)) != "loadstring" &&
00315                std::string(lua_tostring(pt_state, -2)) != "module"    &&
00316                std::string(lua_tostring(pt_state, -2)) != "newproxy" &&
00317                std::string(lua_tostring(pt_state, -2)) != "next" &&
00318                std::string(lua_tostring(pt_state, -2)) != "pairs" &&
00319                std::string(lua_tostring(pt_state, -2)) != "pcall" &&
00320                std::string(lua_tostring(pt_state, -2)) != "rawequal" &&
00321                std::string(lua_tostring(pt_state, -2)) != "rawget" &&
00322                std::string(lua_tostring(pt_state, -2)) != "rawset" &&
00323                std::string(lua_tostring(pt_state, -2)) != "require" &&
00324                std::string(lua_tostring(pt_state, -2)) != "select" &&
00325                std::string(lua_tostring(pt_state, -2)) != "setfenv" &&
00326                std::string(lua_tostring(pt_state, -2)) != "setmetatable" &&
00327                std::string(lua_tostring(pt_state, -2)) != "unpack" &&
00328                std::string(lua_tostring(pt_state, -2)) != "xpcall";
00329       }
00330       else if(nValueType == LUA_TTABLE) {
00331          if(nKeyType == LUA_TNUMBER) {
00332             return true;
00333          }
00334          else if(nKeyType == LUA_TSTRING) {
00335             return 
00336                std::string(lua_tostring(pt_state, -2)) != "_G"        &&
00337                std::string(lua_tostring(pt_state, -2)) != "coroutine" &&
00338                std::string(lua_tostring(pt_state, -2)) != "debug"     &&
00339                std::string(lua_tostring(pt_state, -2)) != "io"        &&
00340                std::string(lua_tostring(pt_state, -2)) != "os"        &&
00341                std::string(lua_tostring(pt_state, -2)) != "package";
00342          }
00343       }
00344       return false;
00345    }
00346 
00347    /****************************************/
00348    /****************************************/
00349    
00350 }
```

---

Generated on 10 Jul 2018 for ARGoS by 
 1.6.1
